# Human lactate dehydrogenase A undergoes allosteric transitions under pH conditions inducing the dissociation of the tetrameric enzyme

**DOI:** 10.1042/BSR20212654

**Published:** 2022-01-28

**Authors:** Alberto Pietro Pasti, Valentina Rossi, Giuseppina Di Stefano, Maurizio Brigotti, Alejandro Hochkoeppler

**Affiliations:** 1 Department of Pharmacy and Biotechnology, University of Bologna, Viale Risorgimento 4, Bologna 40136, Italy; 2 Department of Experimental, Diagnostic and Specialty Medicine, University of Bologna, via S. Giacomo 14, Bologna 40126, Italy; 3 CSGI, University of Florence, via della Lastruccia 3, Sesto Fiorentino 50019, Firenze, Italy

**Keywords:** allosteric regulation, enzyme kinetics, lactate dehydrogenase, lactic acid, liver

## Abstract

The aerobic energetic metabolism of eukaryotic cells relies on the glycolytic generation of pyruvate, which is subsequently channelled to the oxidative phosphorylation taking place in mitochondria. However, under conditions limiting oxidative phosphorylation, pyruvate is coupled to alternative energetic pathways, e.g. its reduction to lactate catalyzed by lactate dehydrogenases (LDHs). This biochemical process is known to induce a significant decrease in cytosolic pH, and is accordingly denoted lactic acidosis. Nevertheless, the mutual dependence of LDHs action and lactic acidosis is far from being fully understood. Using human LDH-A, here we show that when exposed to acidic pH this enzyme is subjected to homotropic allosteric transitions triggered by pyruvate. Conversely, human LDH-A features Michaelis–Menten kinetics at pH values equal to 7.0 or higher. Further, citrate, isocitrate, and malate were observed to activate human LDH-A, both at pH 5.0 and 6.5, with citrate and isocitrate being responsible for major effects. Dynamic light scattering (DLS) experiments revealed that the occurrence of allosteric kinetics in human LDH-A is mirrored by a consistent dissociation of the enzyme tetramer, suggesting that pyruvate promotes tetramer association under acidic conditions. Finally, using the human liver cancer cell line HepG2 we isolated cells featuring cytosolic pH equal to 7.3 or 6.5, and we observed a concomitant decrease in cytosolic pH and lactate secretion. Overall, our observations indicate the occurrence of a negative feedback between lactic acidosis and human LDH-A activity, and a complex regulation of this feedback by pyruvate and by some intermediates of the Krebs cycle.

## Introduction

Lactic acid is an important component of the energetic metabolism, in both prokaryotic and eukaryotic organisms. Among prokaryotes, lactic acid bacteria share the competence in the generation of lactate as a fermentative end product, albeit being members of different genera [1]. The phenotype of lactic acid bacteria relies on lactate dehydrogenases (LDHs), responsible for the reduction of pyruvate to lactate according to a variety of mechanisms, exerted by NADH-dependent or -independent enzymes [2], yielding l- or d-lactate. Bacterial d-lactate dehydrogenases are homodimeric [3–5] or homotetrameric enzymes [6], featuring Michaelis–Menten [7] or cooperative kinetics [8]. Similarly, l-lactate dehydrogenases isolated from bacterial sources are homodimeric [9] or homotetrameric enzymes [2], with representatives featuring allosteric transitions and homotropic or heterotropic activation, e.g. by pyruvate and fructose-1,6-bisphosphate (FBP), respectively [10].

Among eukaryotes, the presence of tetrameric l-LDHs in vertebrates was early detected [11], with d-LDHs thought to be exclusive of eukaryotic invertebrates [12]. However, it was quite recently recognized that genes coding for d-LDHs do occur in vertebrates [13], therefore suggesting that the two stereospecific forms of LDHs can be expressed in the same eukaryotic organism. Concerning the l-LDHs of vertebrates, two major isoforms were long ago identified, i.e. the heart (H, LDH-B) and the muscle (M, LDH-A) enzymes [14,15]. The H form is more abundant in aerobic tissues, and is supposed to mainly act in the oxidation of lactate. The M form is mainly produced in tissues facing transient hypoxia, such as white muscle fibers, where it is devoted to the reduction of pyruvate. In addition, the X form (LDH-C) was isolated from mammalian spermatozoa [16], and it was detected in mitochondria [17].

Physiologically speaking, the relevance of lactate in energetic metabolism is linked to conditions limiting oxidative phosphorylation. This indeed occurs in tissues subjected to intense exercise, whose cells face transient hypoxia and feature a 16-fold increase in the lactate/pyruvate ratio when compared with cells of resting tissues [18]. However, it was shown that lactate can be produced in fully aerobic tissues [19,20], and it was proposed that lactate does always represent the end product of glycolysis [21]. According to this view, the cytosolic lactate produced under fully aerobic conditions is transferred to the mitochondrial intermembrane space, where it undergoes oxidation to pyruvate, which is finally committed to the Krebs cycle [21]. Therefore, any situation limiting the oxidative consumption of pyruvate (not necessarily oxygen limitation) would imply an increase in lactate level, a condition which is known to occur in cancer cells [22]. Remarkably, it has been demonstrated that silencing or inhibiting LDH-A in human B-lymphoid cells does induce cell death via oxidative stress [23]. Moreover, it was also shown that pyruvate counteracts the inhibition exerted by metformin on cancer cells proliferation [24]. This effect triggered by pyruvate is linked to the action of LDH, which is essential to maintain the NAD^+^/NADH molar ratio within values appropriate for cells growth and duplication [24].

Under both physiological and pathological conditions, the occurrence of an energetic metabolism dominated by the glycolytic generation of lactate can be associated with a decrease in intracellular pH. It should be noted that the generation of lactate by LDH does not induce acidification *per se*, the reduction of pyruvate at the expense of NADH + H^+^ being a proton-consuming reaction. Lactic acidosis represents instead the outcome of a net release of H^+^ from ATP hydrolysis under hypoxic or anoxic environments [25].

A peculiar situation is usually detected in cancer cells, whose energetic metabolism is committed to glycolysis and lactate release, and whose pH_i_ is nevertheless higher than in normal cells [26]. The maintenance of high pH_i_ in cancer cells is linked to a repertoire of biochemical factors, the action of which is increased over the levels observed under physiological conditions [26]. This phenotype of cancer cells is presumably related to the necessity to preserve the activity of LDH-A, featuring a pH-sensitivity related to post-translational modifications [27]. This is in contrast with the situation observed in normal cells, whose pH_i_ can be induced to decrease by transient/prolonged hypoxia. When considering cells facing prolonged fatigue and hypoxia, the activity of LDH-A is essential for the energetic metabolism. However, the decrease in pH_i_ linked to hypoxic glycolysis and ATP consumption could trigger a parallel decrease in LDH-A activity, depressing in turn the energetic potential of lactate generation. To investigate this point in detail, we used human LDH-A as a model system. Accordingly, we report here on the activity of human LDH-A as a function of pH, and on the occurrence of allosteric transitions under acidic pH conditions. In addition, the identification of human LDH-A effectors and the characterization of enzyme stability over a wide pH interval are also presented, along with the determination of enzyme activity in human cells, from the liver cancer cell line HepG2, subjected to the acidification of cytosol.

## Materials and methods

### Materials

All reagents were purchased from Merck Millipore (St. Louis, MO, U.S.A.).

### Human LDH

LDH (EC 1.1.1.27) from human liver (LDH-A, enzyme suspension in 3.1 M ammonium sulfate, Lot 04B2913) was obtained from Lee Biosolutions (Maryland Heights, MO, U.S.A.), and recombinant human LDH-A (Lots 118M4133V and 94722) was purchased from Merck Millipore. Appropriate dilutions of the natural liver enzyme were prepared immediately before their use. Recombinant LDH-A was dialyzed against 10 mM Hepes (pH 7.5), concentrated to approximately 10 mg/ml with an Amicon ultrafiltration cell equipped with a YM100 membrane, and aliquots of the concentrated enzyme solution were stored at −20°C until use.

### Activity assays

The enzyme-catalyzed reduction of pyruvate was assayed by determining the decrease in absorbance at 340 nm related to the oxidation of β-NADH. The absorption coefficient of β-NADH at 340 nm was assumed to be equal to 6.22 × 10^3^ M^−1^ cm^−1^ [28]. All the assays were performed at 25 ± 0.1°C using a Cary 300 Bio spectrophotometer. Reactions were started by enzyme addition. To analyze enzyme kinetics as a function of pH, a universal buffer (containing Mes, Mops, and Tris, 25 mM each) was used [29]. The enzyme kinetic parameters were determined under different conditions with the Levenberg–Marquardt algorithm in SigmaPlot 14 (Systat Software, San Josè, CA, U.S.A.). Protein concentration was assayed according to Bradford [30].

To assess the effect, if any, triggered by the intermediates of Krebs cycle on LDH-A activity, stock solutions of each compound to be tested were prepared in distilled H_2_O supplemented with the appropriate volume of NaOH to obtain pH 5.0. The pH of each stock solution accordingly prepared was checked with litmus paper. An identical procedure was used to prepare stock solutions at pH 5.0 of glutamate, aspartate, glutarate, and acetate. Particular care was taken with oxaloacetate (known to be unstable), the stock solutions of which were prepared immediately before use.

### Analysis of LDH-A dissociation equilibria

To evaluate the dissociation of LDH-A tetramer, we assayed initial velocities of pyruvate reduction as a function of enzyme concentration, over pH values ranging from 5.0 to 7.0. Under conditions of zero-order kinetics (0.1 mM pyruvate, 125 µM β-NADH), enzyme activity was found to exponentially depend on LDH-A concentration. To interpret this, the LDH-A tetramer was assumed to undergo two consecutive dissociation equilibria (eqn 1):

(1) $$T\overset{K_{D1}}{⇋}2D\overset{K_{D2}}{⇋}4M$$

Accordingly, the total concentration of enzyme subunits can be expressed as:

(2) $$\left[S_{t}]\;=\;\sqrt{K_{D2\;}\sqrt{K_{D1\;}[T]}\;}+\;2\sqrt{K_{D1}\;[T]\;}+\;4\;[T\right]$$

For each pH considered, the discrete values of activity detected in the presence of known concentrations of total subunits (*S*_t_) were interpreted as the output of the catalytic action exclusively exerted by LDH-A tetramer. Given a particular [*S*_t_], the fraction of enzyme tetramer was estimated by solving (eqn 2) expressed in implicit form. To this aim, we used the implicit equation solver in SigmaPlot to determine the best fit to the experimental observations obtained at each pH value.

### Dynamic light scattering

Dynamic light scattering (DLS) experiments were performed with a Malvern Panalytical (Malvern, U.K.) Zetasizer Nano ZS system. All the measurements were recorded at 25°C using solutions previously filtered with 0.2-µm filters. Scattering was evaluated at an angle of 173 degrees. Each individual observed value of enzyme diameter represents the average output of three groups of consecutive determinations. Raw data were analyzed with the Zetasizer software (Malvern Panalyticals), release 7.11, and the main relevant peaks accordingly identified were further inspected using the Fityk program [31]. By this means, the area of each peak was normalized to 1, and then deconvoluted into a set of Gaussian distributions, with each component interpreted as a homogeneous subpopulation of the enzyme ensemble.

### HepG2 cells

Cells from the human liver cancer cell line HepG2 were grown in T-75 flasks at a density equal to 1–2 × 10^5^/cm^2^, in DMEM. Flasks were maintained at 37°C in a 5% CO_2_ humidified incubator. To evaluate lactate levels, cells were collected two to four culture cycles after thawing. The fluorescent probe BCECF (2′,7′-Bis-(2-carboxyethyl)-5-(6)-carboxyfluorescein, acetoxymethyl ester) was used to determine the cytosolic pH of cells. To this aim, cells were washed twice with HBSS (pH 7.3), incubated with 5 µM BCECF for 30 min at room temperature under mild shaking, and finally washed twice with HBSS (pH 7.3). Intracellular acidosis was induced by washing and incubating cells in HBSS medium (devoid of bicarbonate and supplemented with Hepes and Mes buffers, 10 mM each) at pH 6.5. Control samples were prepared with cells washed and incubated in HBSS medium at pH 7.3. The fluorescence of BCECF was determined using a PerkinElmer (Waltham, MA, U.S.A.) Enspire microplate reader, exciting samples at 440 or 490 nm, and evaluating emission at 535 nm. The calibration of BCECF fluorescent response to pH was obtained by exposing cells to a medium containing 0.8 mM MgSO_4_, 1.8 mM CaCl_2_, 140 mM KCl, 5 mM glucose, supplemented with Mes and Hepes buffers (10 mM each), and conditioned at pH values ranging from 5.0 to 8.0. After addition of 10 µM nigericin, cells were incubated for 10 min at room temperature under mild shaking, and the fluorescence of BCECF was finally determined under the same conditions used to estimate cytosolic pH.

### Lactate assay

The concentration of lactate released by rabbit skeletal muscle cells was determined using two independent methods, relying on colorimetric [32,33] or enzymatic [34] detection. Cells (5 × 10^5^/well) were seeded in six-well plates and left to adhere overnight. After adhesion, growth medium was replaced with 1 ml of HBSS (at pH 6.5 or 7.3) containing 20 mM glucose and cells were allowed to equilibrate for 3 h in a 37°C incubator. After 3 h of further incubation at 37°C, HBSS medium was collected from each well to evaluate lactate levels. Duplicate experiments were performed. The enzymatic assay consisted in the addition of appropriate volumes of samples to a reaction mixture containing (final concentrations) 125 mM glycine, 40 mM hydrazine, 10 mM EDTA, 2.5 mM β-NAD^+^, 105 nM rabbit LDH, pH 9.0. The increase in absorbance at 340 nm related to the oxidation of lactate and the concomitant reduction of β-NAD^+^ was observed for 3 h, a time interval sufficient to approximate reaction equilibrium. To estimate lactate concentration in samples, a calibration curve was determined by a series of assays performed in the presence of known concentrations of sodium lactate.

## Results

### Kinetics of recombinant and natural human LDH-A as a function of pH

To test the effect of pH on the catalytic action of human LDH-A (UniProtKB code P00338), we assayed the activity of both recombinant and natural enzymes over a wide pH interval, i.e. 5.0–8.0. In particular, we determined the initial velocities of reactions catalyzed by LDH-A as a function of pyruvate concentration, in the presence of a constant β-NADH concentration (125 µM).

**Figure 1:**
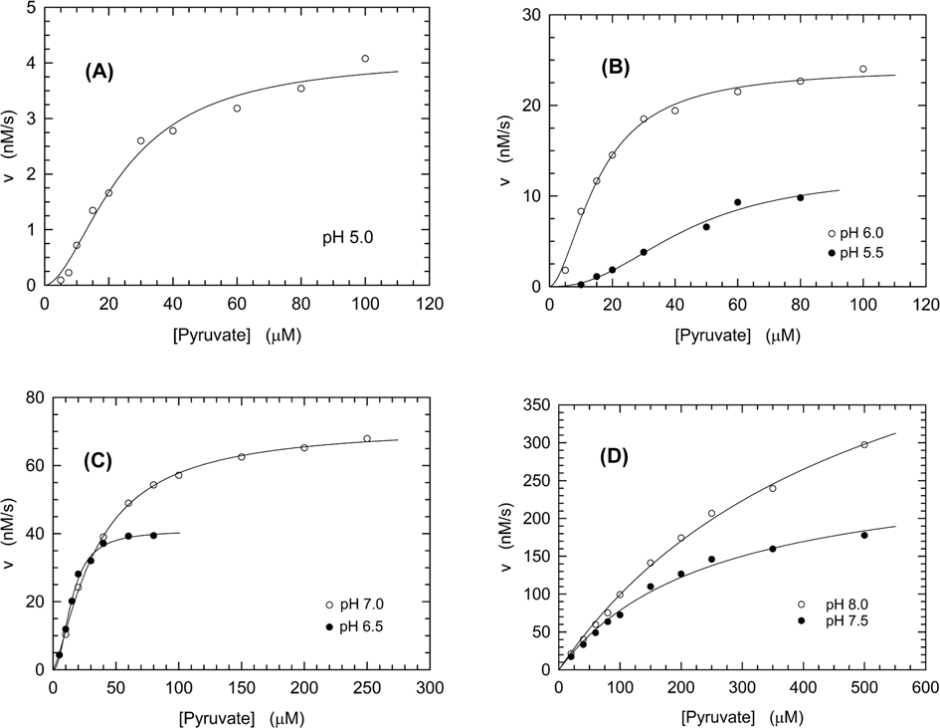
Kinetics of pyruvate reduction catalyzed by recombinant human LDH-A (**A–D**) Initial reaction velocities were assayed over a pH interval ranging from 5.0 to 8.0, in the presence of 2.45 nM LDH-A tetramer (9.8 nM subunits), 125 µM β-NADH, and a universal buffer (containing Mes, Mops, and Tris, 25 mM each). The dependence of initial reaction velocities on pyruvate concentration at pH 5.0 (A, empty circles), pH 5.5 and 6.0 (B, filled and empty circles, respectively), pH 6.5 and 7.0 (C, filled and empty circles, respectively), pH 7.5 and 8.0 (D, filled and empty circles, respectively) are shown. The continuous lines represent the best fit of the Hill (pH 5.0–7.0) or the Michaelis–Menten (pH 7.5–8.0) equation to the experimental observations.

First, we investigated the performance of the recombinant enzyme, which was expressed in *Escherichia coli* and is therefore devoid of any post-translational modification. Remarkably, when the activity assays were carried out at pH values from 5.0 to 7.0, we observed sigmoidal kinetics (Figure 1A–C). Moreover, the *K*_0.5_ of LDH-A for pyruvate was not severely affected by pH over the 5.0–7.0 interval, whereas *V*_max_ was observed to monotonically increase as a function of pH (Table 1), accounting for a parallel increase in the enzyme catalytic efficiency (Figure 1A–C). It is also interesting to note that the values of the Hill coefficient were almost constant and approximately equal to 1.7–2.0 when determined at pH values from 5.0 to 6.5, whereas at pH 7.0 the Hill coefficient was estimated to decrease to 1.34 ± 0.05 (Table 1). Interestingly, when LDH-A activity was assayed at pH 7.5 or 8.0, the enzyme was observed to obey Michaelis–Menten kinetics (Figure 1D), featuring high values for both *K*_m_ and *V*_max_ (Table 1). Nevertheless, the sharp increase in *K*_m_ occurring at pH 7.5–8.0 limits the catalytic efficiency of LDH-A, which features a maximum at pH 6.5 (Table 1).

**Table 1:** Kinetic parameters of recombinant and natural human LDH

pH	*K*_0.5_ (μM)	*V*_max_ (nM/s)	Hill
Human LDH-A, recombinant			
5.0	24.7 ± 3.1	4.1 ± 0.3	1.78 ± 0.27
5.5	43.4 ± 8.7	12.5 ± 2.2	2.32 ± 0.49
6.0	15.7 ± 1.0	24.2 ± 0.8	1.73 ± 0.18
6.5	14.9 ± 0.6	41 ± 1	2.12 ± 0.18
7.0	34.7 ± 1.2	72 ± 1	1.34 ± 0.05
7.5	252 ± 30	276 ± 16	-
8.0	500 ± 44	596 ± 33	-
Human LDH-A, natural			
5.0	56 ± 4	19 ± 1	2.01 ± 0.28
5.5	34 ± 3	87 ± 4	1.65 ± 0.19
6.0	43 ± 4	82 ± 4	1.51 ± 0.21
6.5	82 ± 11	211 ± 14	1.13 ± 0.08
7.0	95 ± 9	296 ± 11	-
7.5	210 ± 17	389 ± 15	-
8.0	1083 ± 359	1306 ± 330	-

**Figure 2:**
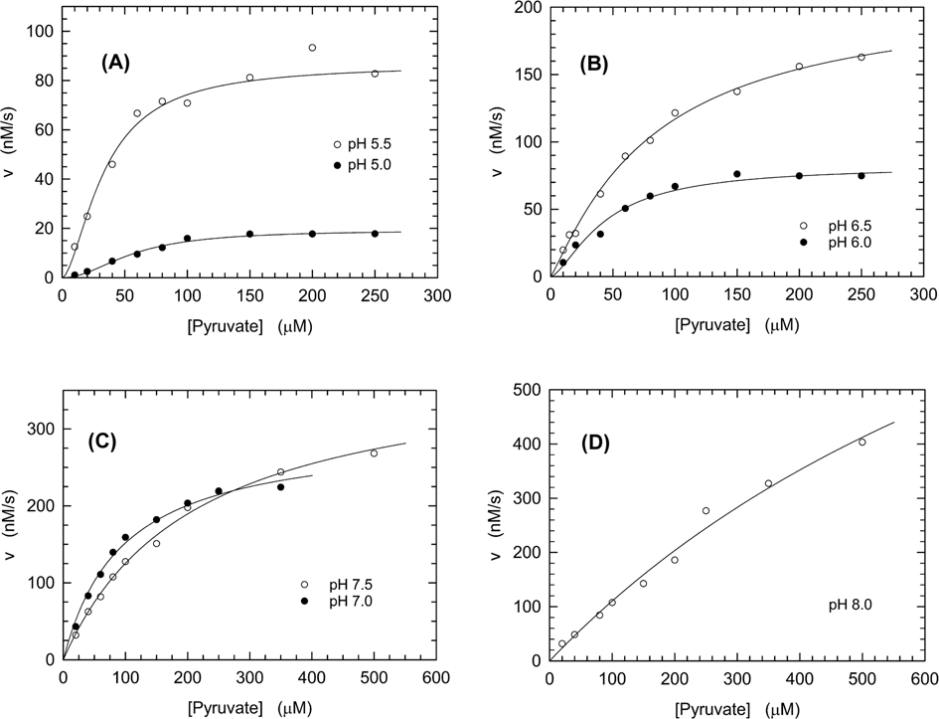
Kinetics of pyruvate reduction catalyzed by natural human LDH-A (**A–D**) Initial reaction velocities were assayed over a pH interval ranging from 5.0 to 8.0, in the presence of 1.2 nM LDH-A tetramer (4.8 nM subunits), 125 µM β-NADH, and a universal buffer (containing Mes, Mops, and Tris, 25 mM each). The dependence of initial reaction velocities on pyruvate concentration at pH 5.0 and 5.5 (A, filled and empty circles, respectively), pH 6.0 and 6.5 (B, filled and empty circles, respectively), pH 7.0 and 7.5 (C, filled and empty circles, respectively), pH 8.0 (D, empty circles) are shown. The continuous lines represent the best fit of the Hill (pH 5.0–6.5) or the Michaelis–Menten (pH 7.0–8.0) equation to the experimental observations.

The Michaelis–Menten or the Hill equation was fitted to the experimental observations shown in Figures 1 and 2. The indicated standard deviations of the enzyme parameters are derived from the fitting of the relevant equation to the experimental observations.

We then tested the natural (isolated from liver) human LDH-A, under the same conditions used to assay the recombinant enzyme. Overall, the kinetic constants determined for the natural enzyme as a function of pH are in satisfactory agreement with those estimated for recombinant LDH-A. In particular, a parallel increase in pH and enzyme activity was also observed for the natural enzyme (Figure 2), the *K*_0.5_ and *V*_max_ of which were observed to be affected by pH with a dependence similar to that featured by the recombinant enzyme (Table 1). Nevertheless, the *V*_max_ values determined for the natural enzyme are significantly higher than those estimated for recombinant LDH-A, and the transition from cooperative to Michaelis–Menten kinetics was found to occur for the natural enzyme at pH 7.0 (Table 1), whereas at this pH value recombinant LDH-A does obey sigmoidal kinetics (Table 1).

Substrate inhibition was reported to affect LDHs isolated from different species [35–39], with both β-NADH and pyruvate capable to exert this action on the human enzyme [38]. Moreover, it was demonstrated that when an excess of pyruvate is provided to cultured human cells a significant inhibition of LDH activity occurs both *in vivo* and *ex vivo* [40]. Remarkably, we also observed that pyruvate can inhibit LDH-A activity, with this inhibition detected at pH values ranging from 5.0 to 7.0 for the recombinant enzyme (Supplementary Figure S1). It should be noted that this implies the occurrence of substrate inhibition at the same pH values inducing sigmoidal kinetics (Figure 1 and Supplementary Figure S1), suggesting a common molecular determinant for these experimental outcomes. Substrate inhibition by pyruvate was also observed when the activity of natural human LDH-A was assayed (Supplementary Figure S2), albeit to a lower extent when compared with the inhibition exerted on the recombinant enzyme (Supplementary Figures S1 and S2).

### Activation of LDH-A by Krebs cycle intermediates

**Figure 3:**
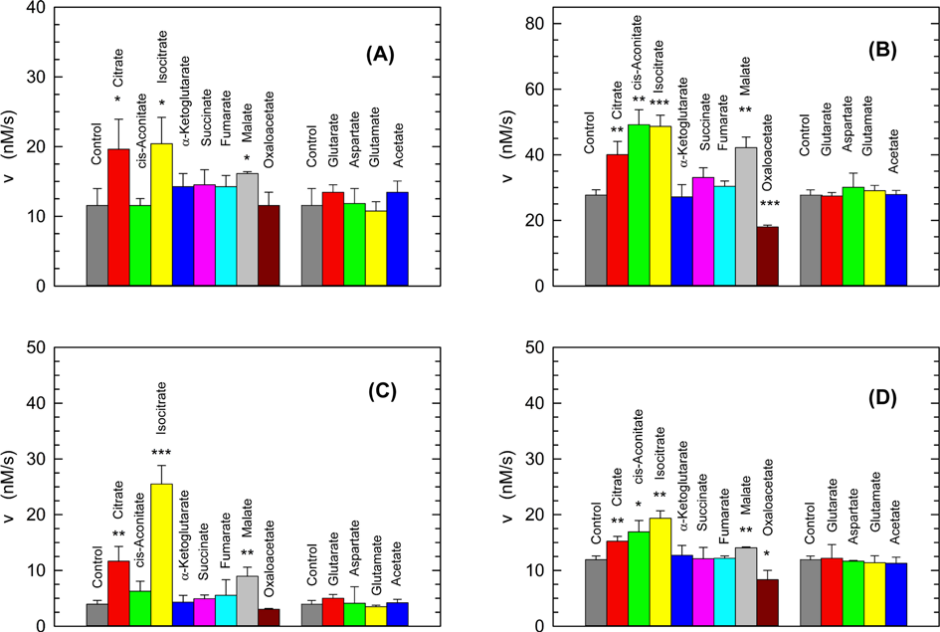
Effect of the Krebs cycle intermediates on the activity of human LDH-A (**A**,**B**) Initial reaction velocities determined at pH 5.0 (A) and 6.5 (B) in the presence of 8 nM recombinant human LDH-A tetramer (32 nM subunits), 125 µM β-NADH, and 100 µM pyruvate (control, gray bars). The LDH-A activities observed using reaction mixtures supplemented with the indicated compounds at 2.5 mM are also shown. The reported values of initial reaction velocity are the average of three samples. The error bars represent standard deviation (*n*=3). Experimental mean values were compared by Student’s *t* test (***, ** and * indicate *P*<0.001, *P*<0.01, *P*<0.05, respectively). (**C,D**) Initial reaction velocities determined at pH 5.0 (C) and 6.5 (D) in the presence of 0.3 nM natural human LDH-A tetramer (1.2 nM subunits), 125 µM β-NADH, and 100 µM pyruvate (control, gray bars). The LDH-A activities observed using reaction mixtures supplemented with the indicated compounds at 2.5 mM are also shown. The reported values of initial reaction velocity are the average of three samples. The error bars represent standard deviation (*n*=3). Experimental mean values were compared by Student’s *t* test (***, ** and * indicate *P*<0.001, *P*<0.01, *P*<0.05, respectively).

Quite a number of years ago, Fritz reported that rabbit skeletal muscle LDH-A undergoes allosteric transitions [41]. In addition, Fritz tested the action of different intermediates of the Krebs cycle on rabbit LDH-A, and he found that citrate, isocitrate, malate, *cis*-aconitate, fumarate, succinate, and α-ketoglutarate activate the enzyme, with malate and citrate producing the stronger effects [41]. Accordingly, we thought it of interest to assay the effect, if any, of the Krebs cycle intermediates on the activity of human LDH-A. In particular, both recombinant and natural LDH-A were used to determine if their activity was affected by the compounds to be tested, at pH 5.0 and 6.5. Moreover, to avoid the occurrence of any substrate inhibition in these assays the final concentration of pyruvate in the reaction mixtures was limited to 100 µM. When the recombinant enzyme is considered, we observed enzyme activation at pH 5.0 by citrate, isocitrate, and malate (Figure 3A). These three Krebs cycle intermediates were also found to activate recombinant human LDH-A at pH 6.5; in addition, under these conditions *cis*-aconitate and oxaloacetate were observed to exert activation and inhibition of the enzyme, respectively (Figure 3B). Quantitatively speaking, citrate and isocitrate triggered the more pronounced effects at pH 5.0, with the enzyme activity being almost doubled when compared with the level detected in the control reaction mixture (Figure 3A). At pH 6.5, a two-fold increase in activity was induced by isocitrate and *cis*-aconitate, with citrate inducing a significant activation of recombinant LDH-A, albeit to a smaller extent when compared with the level of activation exerted at pH 5.0 (Figure 3B). When the activity assays were performed in the presence of natural LDH-A, we observed activation effects similar to those detected in the presence of the recombinant enzyme. In particular, at pH 5.0 citrate, isocitrate, and malate performed as enzyme activators, with isocitrate responsible for a three-fold increase in activity (Figure 3C). At pH 6.5 the natural enzyme was activated by the same four compounds activating recombinant LDH-A, i.e. citrate, isocitrate, malate, and *cis*-aconitate (Figure 3D); moreover, at pH 6.5 oxaloacetate inhibited both recombinant and natural LDH-A (Figure 3B,D).

### Reaction velocity and LDH-A concentration

**Figure 4:**
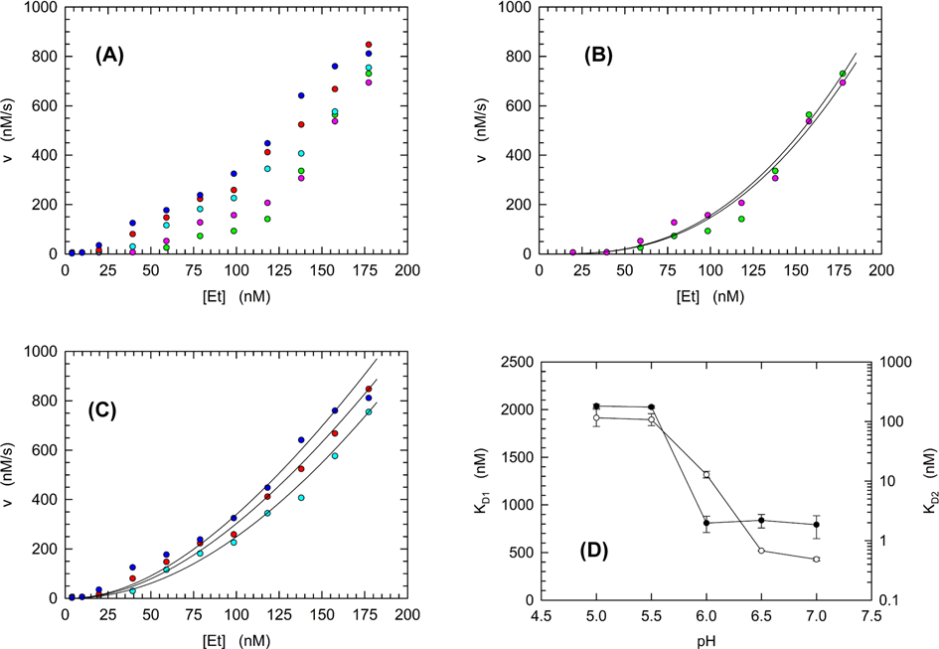
Activity of recombinant human LDH-A as a function of enzyme concentration and pH (**A**) The activity of LDH-A was determined at pH 5.0, 5.5, 6.0, 6.5, and 7.0 (green, pink, cyan, red, and blue circles, respectively). Assays were performed in the presence of 125 µM β-NADH and 100 µM pyruvate. Enzyme concentrations are expressed as the total concentration of subunits per assay. (**B**) Detail of the activity determinations performed at pH 5.0 and 5.5. The continuous lines represent the expected values of activity according to the estimations of *K*_D1_ and *K*_D2_ obtained with the implicit equation solver in SigmaPlot (see ‘Materials and methods’ section). (**C**). Detail of the activity determinations performed at pH 6.0, 6.5, and 7.0. The continuous lines represent the expected values of activity according to the estimations of *K*_D1_ and *K*_D2_ obtained with implicit equation solver in SigmaPlot (see ‘Materials and methods’ section). (**D**) Dependence of *K*_D1_ (empty circles) and *K*_D2_ (filled circles) on pH.

Among the Krebs cycle intermediates competent in activating human LDH-A (Figure 3), citrate is known to bind at the interface of enzyme subunits [42]. This binding could enhance enzyme activity according to different mechanisms: (i) inducing a conformational transition favorable to high catalytic efficiency; (ii) counteracting the dissociation of LDH-A tetramer into dimers and/or monomers featuring low activity, if at all, as it was shown for pig LDH [43]. Concerning the conformational rearrangement, it should be noted that *Lactobacillus casei* LDH undergoes a transition from the T to the R state when sulfate is bound at the enzyme subunits interface [10]. Moreover, the same enzyme was found to maintain the T conformation when nitrate is bound between subunits, indicating that the T to R transition is affected by the type of anion bound by LDH [10]. However, no similar behavior was observed for eukaryotic LDHs, which are conventionally considered to obey Michaelis–Menten kinetics [44]. Rather intriguingly, it was reported for rabbit LDH-A that lowering the enzyme concentration in activity assays corresponds to an exponential decrease in the observed catalytic action [45–47], suggesting a dilution-induced dissociation of the enzyme tetramer. Therefore, we reasoned that the allosteric nature of human LDH-A at acidic pH values might represent the outcome of the dissociation of the enzyme tetramer, with this dissociation counteracted by the binding of pyruvate (Figures 1 and 2). Further, the binding of citrate to human LDH-A could promote the stabilization of the enzyme tetramer, inducing an increase in activity (Figure 3). Accordingly, we thought it of interest to assay LDH-A activity as a function of enzyme concentration, under different pH conditions. As expected for a dilution-induced LDH-A tetramer dissociation, we detected an exponential dependence of activity on total subunits concentration (Figure 4A). In addition, significant differences were observed as a function of pH. Qualitatively speaking, the concentration of total enzyme subunits necessary to reach half of the maximal observed activity did monotonically decrease as pH was increased (Figure 4A). To obtain a better estimation of the mutual dependence between pH and LDH-A dissociation, we used a tetramer–dimer–monomer dissociation model. In particular, an implicit equation describing the dissociation equilibria was fitted to the experimental observations (see ‘Materials and methods’ section). By this means, we estimated the *K*_D_ values at each pH for the dissociation of tetrameric LDH-A (Figure 4B–D). Remarkably, under the lower pH conditions tested (5.0 and 5.5) both *K*_D1_ and *K*_D2_ were determined to feature the highest estimated values, corresponding to ∼2 and 0.1 µM, respectively (Figure 4D). Moreover, the value of *K*_D1_ (accounting for the dissociation of tetramer into dimers) does consistently decrease between pH 5.5 and 6.5, and undergoes a further, albeit modest, decrease at pH 7.0. Accordingly, it is important to note that the tetramer of human LDH-A features significant dissociation at neutral pH, suggesting that the enzyme association forms occurring *in vivo* are significantly controlled by the expression levels of LDH-A.

### Dissociation of LDH-A as a function of pH

**Figure 5:**
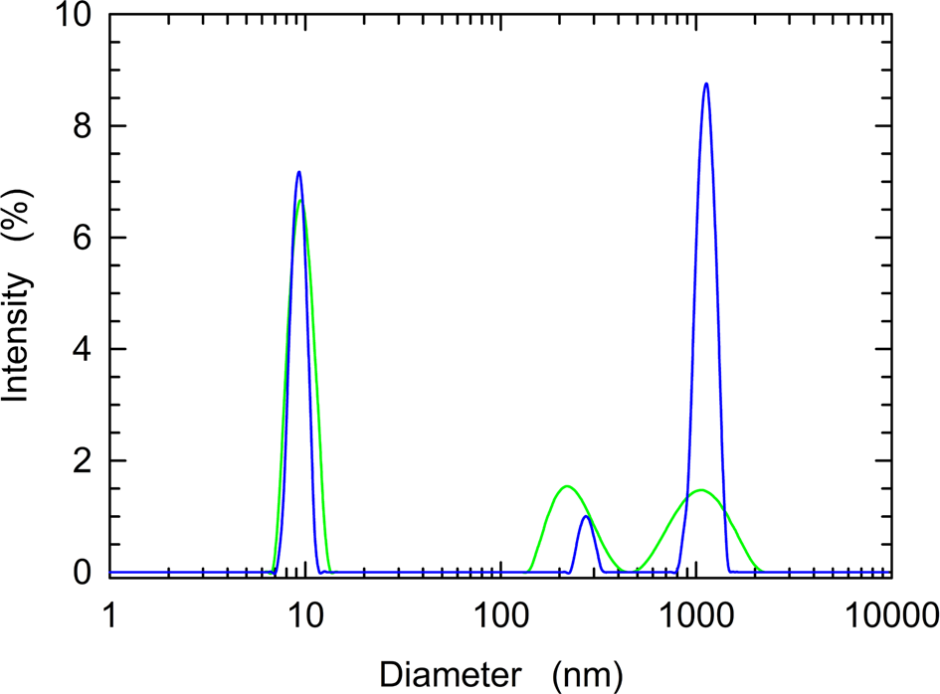
Association forms of recombinant human LDH-A detected at pH 5.0 and 7.5 by DLS DLS measurements were acquired at pH 7.5 (green line) or 5.0 (blue line) using LDH-A solutions containing 7 µM tetrameric enzyme (28 µM subunits).

To further inspect the effect of pH on the dissociation of LDH-A tetramer, we performed DLS experiments over a pH interval ranging from 5.0 to 7.5. First, we prepared for DLS analysis equimolar samples of recombinant LDH-A at pH 5.0 and 7.5. Interestingly, at pH 7.5 enzyme association forms featuring large diameters were detected as minor components, whereas at pH 5.0 the occurrence of large aggregates was clearly observed (Figure 5), suggesting the dissociation of LDH-A tetramer and a consequent aggregation of the dissociated forms.

**Figure 6:**
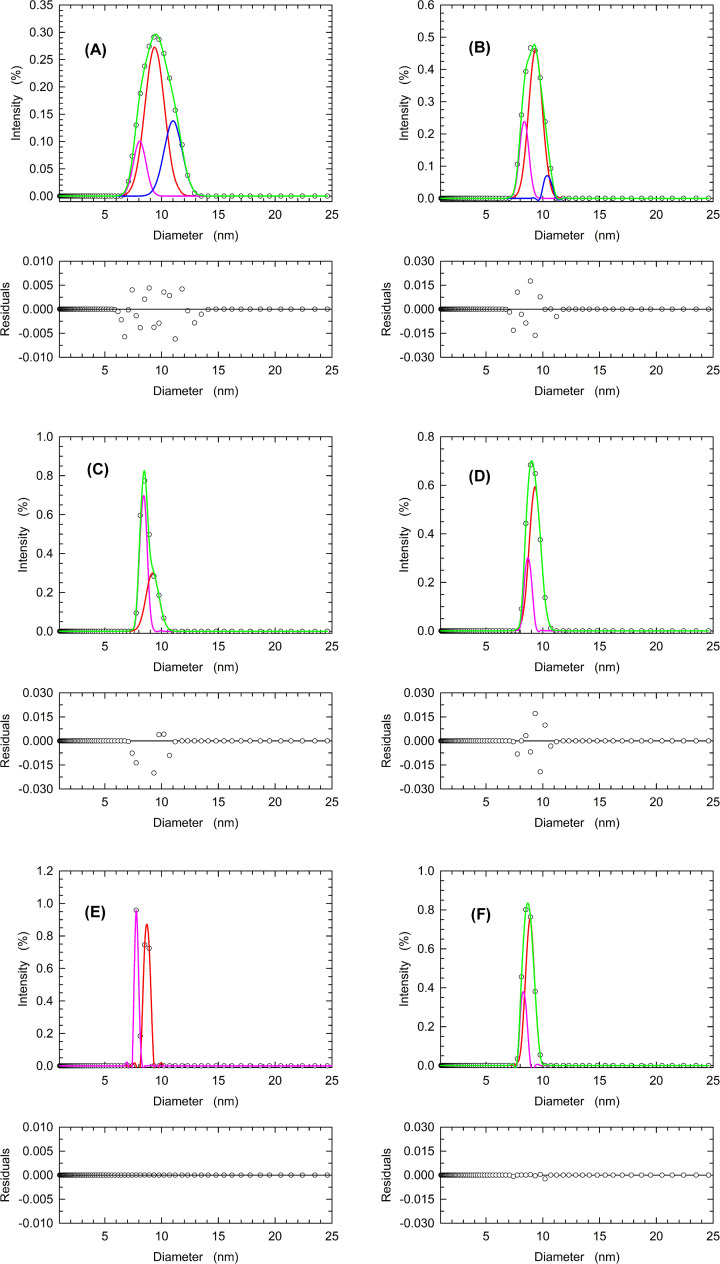
Relative abundance of recombinant human LDH-A tetramer, dimer, and monomer as a function of pH DLS measurements performed at: (**A**) pH 7.5, (**B**) pH 7.0, (**C**) pH 6.5, (**D**) pH 6.0, (**E**) pH 5.5, (**F**) pH 5.0, in the presence of 7 µM tetrameric enzyme (28 µM subunits). The distributions of the observed scattering intensities (empty circles, average of three measurements) were deconvoluted with the Fityk software into an ensemble of Gaussian components (blue, red, and pink lines). The fittings to the experimental observations accordingly obtained are reported with green lines.

The presence of LDH-A tetramer and dissociated forms was then studied in detail with samples prepared at pH values differing by 0.5 units over an interval from 5.0 to 7.5. Moreover, the peaks centered at a diameter value of ∼9–10 nm were further analyzed using the Fityk software [31]. By this means, we deconvoluted each observed peak into Gaussian components representing subpopulations of the enzyme ensemble. When this approach was applied to a 7 µM LDH-A sample at pH 7.5 three main components were detected, featuring diameters equal to 11.01, 9.39, and 8.09 nm which we interpreted as enzyme tetramer, dimer, and monomer, respectively (Figure 6A and Table 2). A similar behavior was observed with an LDH-A sample at pH 7.0, the components of which featured diameters equal to 10.43, 9.39, and 8.36 nm (Figure 6B and Table 2). Remarkably, when the samples at pH 6.5 or lower were analyzed only two enzyme forms were detected (Figure 6C–F and Table 2), with diameters suggesting the presence of dimer and monomer (at pH 6.5 and 6.0) or monomer only (at pH 5.5 and 5.0).

**Table 2:** Effect of pH on the diameters of human LDH-A association forms estimated by DLS experiments

Sample	Diameter (nm)	Area (%)
pH 7.5 7 µM LDH (Holo)	11.01 (T)	27.5
	9.39 (D)	58.9
	8.09 (M)	14.0
pH 7.0 7 µM LDH (Holo)	10.43 (T)	6.3
	9.39 (D)	70.3
	8.36 (M)	24.6
pH 6.5 7 µM LDH (Holo)	9.20 (D)	42.9
	8.40 (M)	57.4
pH 6.0 7 µM LDH (Holo)	9.31 (D)	75.5
	8.69 (M)	24.8
pH 5.5 7 µM LDH (Holo)	8.65 (M)	65.8
	7.76 (M)	36.4
pH 5.0 7 µM LDH (Holo)	8.87 (M)	74.3
	8.30 (M)	26.3

The diameters of different human LDH-A oligomers were estimated by deconvolution of the peaks observed with DLS experiments performed at the indicated pH values in the presence of 7 µM LDH-A tetramer (28 µM subunits). The area of each peak to be deconvoluted was normalized to 1. In addition to the estimated diameters, the area of each component is also reported. T, D, and M indicate the assignment of a detected component to enzyme tetramer, dimer, and monomer, respectively.

**Figure 7:**
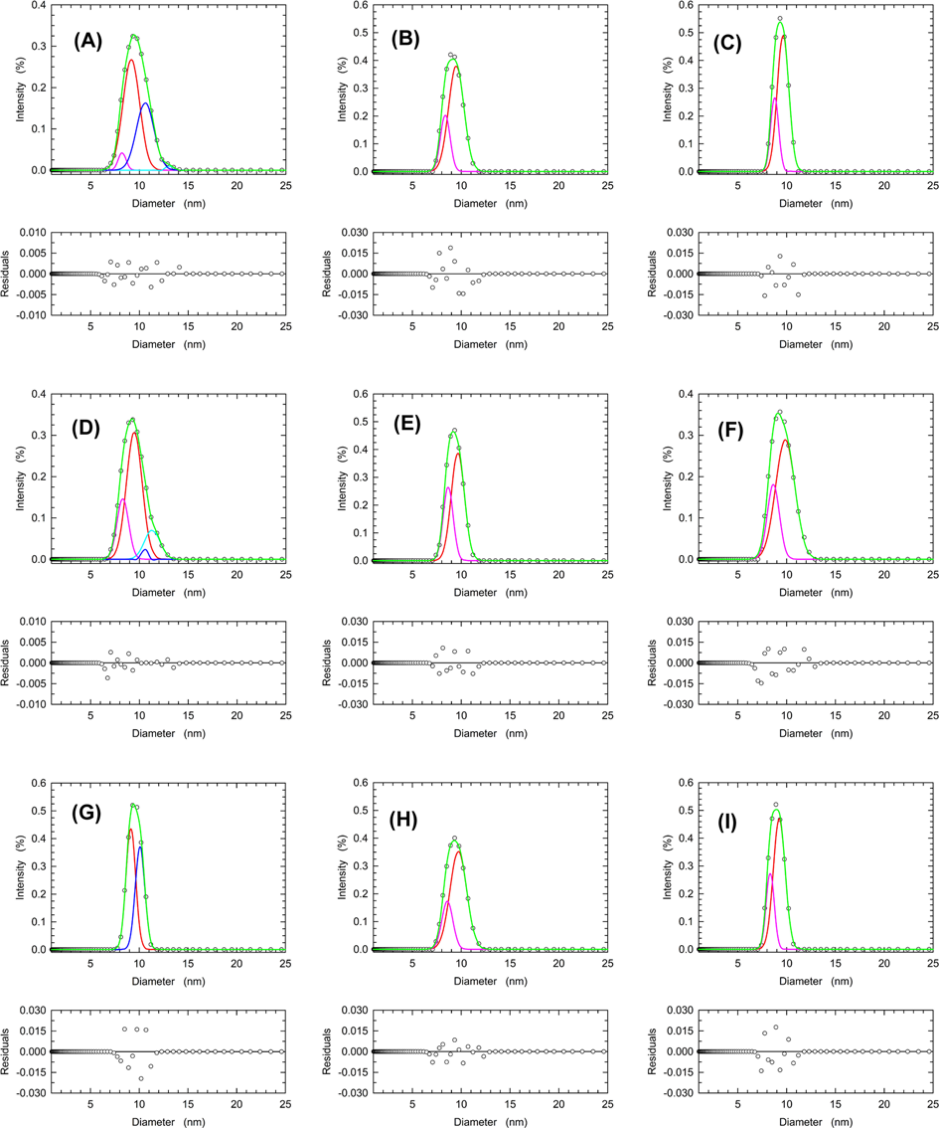
Relative abundance of recombinant human LDH-A tetramer, dimer, and monomer as a function of pH and enzyme concentration DLS measurements performed at pH 7.5 (**A–C**), 6.5 (**D–F**), and 5.0 (**G–I**) in the presence of 8.4 (A,D,G), 4.2 (B,E,H), or 2.1 (C,F,I) µM tetrameric enzyme (33.6, 16.8, and 8.4 µM subunits, respectively). The distributions of the observed scattering intensities (empty circles, average of three measurements) were deconvoluted with the Fityk software into an ensemble of Gaussian components (blue, red, and pink lines). The fittings to the experimental observations accordingly obtained are reported with green lines.

**Figure 8:**
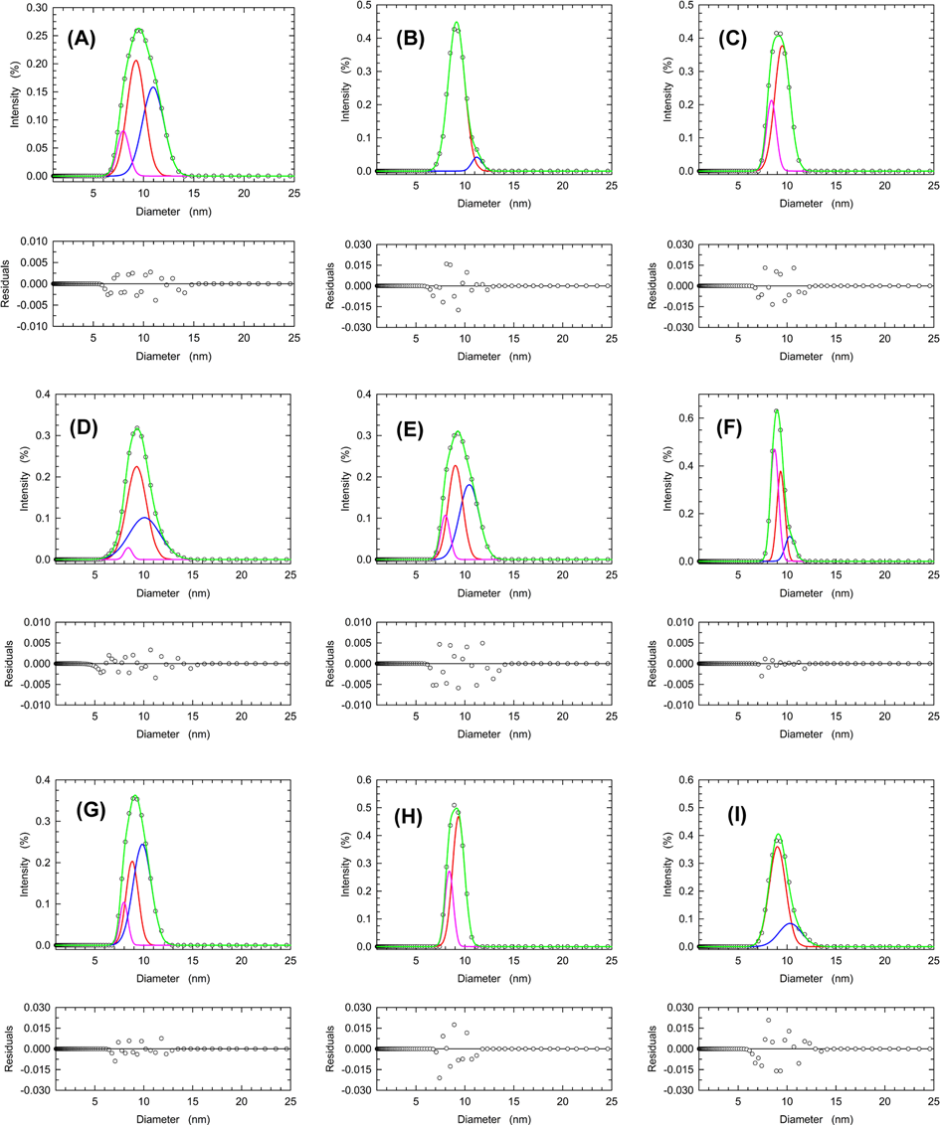
Effect of citrate or NAD^+^/pyruvate on human LDH-A association forms detected at pH 5.0, 6.5, and 7.5 DLS experiments were performed in the presence of 8 µM human LDH-A tetramer (32 µM subunits) at pH 7.5 (**A**,**D**,**G**), 6.5 (**B**,**E**,**H**), or 5.0 (**C**,**F**,**I**), in the absence of any additive (A–C), or in the presence of 2.5 mM citrate (D–F), or NAD^+^/pyruvate (G–I) at 2.5 and 0.5 mM, respectively. The distributions of the observed scattering intensities (empty circles, average of three measurements) were deconvoluted with the Fityk software into an ensemble of Gaussian components (blue, red, and pink lines). The fittings to the experimental observations accordingly obtained are reported with green lines.

We then decided to test the effect of dilution on the dissociation of LDH-A, using samples containing 8.4, 4.2, or 2.1 µM enzyme (Holoenzyme concentration), and prepared at pH 7.5, 6.5, and 5.0. When the higher enzyme concentration is considered, the LDH-A tetramer was detected in the samples at pH 7.5 and 6.5, and pH 5.0 (Figure 7A,D,C and Table 3). However, the LDH-A tetramer was not detected in samples containing 4.2 or 2.1 µM enzyme, independently of pH (Figure 7B,C,E,F,H,I and Table 3). These observations indicate that the assembly of LDH-A tetramer is strongly affected by pH and by the concentration of total subunits. This suggests, in turn, that the allosteric transitions of human LDH-A reported here (Figures 1 and 2) represent the outcome of pyruvate-induced tetramer assembly. Accordingly, we tested by DLS experiments the effect of pyruvate on the distribution of LDH-A association forms at pH 7.5, 6.5, and 5.0. In addition, the action of citrate was also tested under the same conditions. As expected, LDH-A tetramer was detected in control samples (devoid of any additive) at pH 7.5, was found to decrease at pH 6.5 (with enzyme monomer as the major form), and was not observed at pH 5.0 (Figure 8 and Table 4). Remarkably, the presence of 2.5 mM citrate counteracted the dissociation of enzyme tetramer, the presence of which was clearly detected both at pH 6.5 and 5.0 (Figure 8D–F and Table 4). In addition, the presence of pyruvate was observed to favor the assembly of dimer at pH 6.5, and to hinder tetramer dissociation at pH 5.0 (Figure 8G–I and Table 4).

**Table 3:** Effect of pH and enzyme concentration on the diameters of human LDH-A association forms estimated by DLS experiments

Sample	Diameter (nm)	Area (%)
pH 7.5 8.4µM LDH (Holo)	13.18 (T)	2.1
	10.59 (T)	37.0
	9.17 (D)	57.7
	8.23 (M)	4.3
pH 6.5 8.4 µM LDH (Holo)	11. 30 (T)	14.2
	10.52 (T)	2.0
	9.48 (D)	62.0
	8.30 (M)	22.0
pH 5.0 8.4 µM LDH (Holo)	10. 09 (T)	45.8
	9.14 (D)	54.5
pH 7.5 4.2 µM LDH (Holo)	9.49 (D)	75.3
	8.36 (M)	25.3
pH 6.5 4.2 µM LDH (Holo)	9.65 (D)	66.2
	8.66 (M)	34.1
pH 5.0 4.2 µM LDH (Holo)	9.70 (D)	75.8
	8.58 (M)	24.5
pH 7.5 2.1 µM LDH (Holo)	9.63 (D)	73.5
	8.77 (M)	27.6
pH 6.5 2.1 µM LDH (Holo)	9.86 (D)	72.2
	8.64 (M)	28.2
pH 5.0 2.1 µM LDH (Holo)	9.24 (D)	72.1
	8.32 (M)	28.7

The diameters of different human LDH-A oligomers were estimated by deconvolution of the peaks observed with DLS experiments performed at the indicated pH values in the presence of 8.4, 4.2, or 2.1 µM LDH-A tetramer (33.6, 16.8, or 8.4 µM subunits). The area of each peak to be deconvoluted was normalized to 1. In addition to the estimated diameters, the area of each component is also reported. T, D, and M indicate the assignment of a detected component to enzyme tetramer, dimer, and monomer, respectively.

**Table 4:** Effect of citrate and NAD^+^/pyruvate on the diameters of human LDH-A association forms estimated by DLS experiments

Sample	Diameter (nm)	Area (%)
Control pH 7.5 8 µM LDH (Holo)	10.96 (T)	42.4
	9.25 (D)	45.9
	7.98 (M)	12.0
Control pH 6.5 8 µM LDH (Holo)	11.25 (T)	5.7
	9.14 (D)	94.6
Control pH 5.0 8 µM LDH (Holo)	9.52 (D)	73.9
	8.40 (M)	26.5
Citrate pH 7.5 8 µM LDH (Holo)	10.07 (T)	40.3
	9.27 (D)	56.9
	8.40 (M)	2.9
Citrate pH 6.5 8 µM LDH (Holo)	10.45 (T)	45.6
	9.04 (D)	42.3
	8.00 M)	12.7
Citrate pH 5.0 8 µM LDH (Holo)	10.32 (T)	13.2
	9.36 (D)	37.6
	8.72 (M)	49.4
NAD/Pyr pH 7.5 8 µM LDH (Holo)	9.84 (T)	57.2
	8.82 (D)	32.2
	7.97 (M)	10.8
NAD/Pyr pH 6.5 8 µM LDH (Holo)	9.35 (D)	72.0
	8.39 (M)	29.0
NAD/Pyr pH 5.0 8 µM LDH (Holo)	10.32 (T)	24.2
	9.02 (D)	76.3

The distribution of the enzyme ensemble among different association forms was evaluated at the indicated pH values with LDH-A solutions supplemented with 2.5 mM citrate or with NAD^+^/pyruvate (2.5 and 0.5 mM, respectively). Control samples devoid of any additive were also analyzed. T, D, and M indicate the assignment of a detected component to enzyme tetramer, dimer, and monomer, respectively. In addition to the estimated diameters, the area of each component is also indicated.

Finally, it is important to note that the diameters estimated for the different LDH-A association forms appear to be sufficiently invariant to consider them consistent. Indeed, by taking into account all the estimated values (Tables 2-4) the mean diameter for LDH-A tetramer, dimer, and monomer were calculated as equal to 10.55 ± 0.46, 9.34 ± 0.25, and 8.38 ± 0.29, respectively.

### Cytosolic pH of HepG2 cells and LDH-A activity *in vivo*

**Figure 9:**
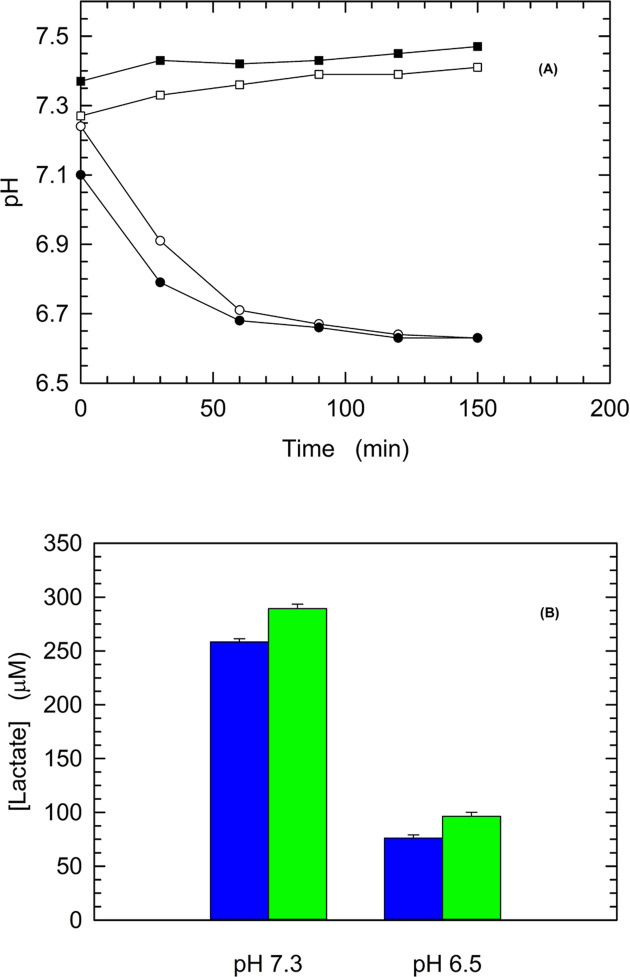
Cytosolic pH of HepG2 cells and generation of lactate (**A**) Cytosolic pH of HepG2 cells after their transfer from HBSS medium at pH 7.3 to the same medium (squares) or to HBSS buffered at pH 6.5 (circles). The values of cytosolic pH were determined using the fluorescent probe BCECF (see ‘Materials and methods’ section and Supplementary Figure S3). Experiments were performed in duplicate (empty and filled symbols). (**B**) Concentration of the lactate released by cells after 3 h of residence in HBSS medium at pH 7.3 or 6.5. Lactate was determined with a colorimetric (blue bars) or with an enzymatic (green bars) analytical procedure, using two independent samples for each determination. Error bars represent the standard deviation (*n*=2).

The dissociation of LDH-A tetramer induced by acidic pH conditions suggests that the enzyme activity can significantly decrease when pH diminishes, especially under conditions of low pyruvate concentration (Figures 1 and 2). Accordingly, we reasoned that this could translate into a moderate lactate production by cells the cytosolic pH of which faces a decrease from the physiological value, which is considered equal to 7.2 [26]. Therefore, we tested by two different analytical methods the concentration of lactate secreted by human HepG2 cells, which are known to predominantly express LDH-A [48]. First, to estimate the cytosolic pH we quantitatively determined the effect of pH on the fluorescence *in vivo* of the probe BCECF (see ‘Materials and methods’ section and Supplementary Figure S3). Then, HepG2 cells previously cultured in DMEM were transferred to HBSS medium buffered at pH 7.3 or 6.5. The time-course of cytosolic pH after transferring the cells from DMEM to HBSS is shown in Figure 9A. According to the observed kinetics, we decided to quantitate the lactate released by HepG2 cells 3 h after their transfer to HBSS medium. Independently of the analytical procedure used, the concentration of lactate released into the external medium by cells featuring the lower cytosolic pH was found to decrease three-fold when compared with the concentration of lactate secreted by cells exposed to HBSS at pH 7.3 (Figure 9B). We interpret this observation as the outcome of the dissociation *in vivo* of LDH-A tetramer, with the enzyme dissociation forms characterized by a low, if at all, catalytic action. It should indeed be considered that 6.5 is the optimal pH for enzyme activity *in vitro* (Table 1), and we accordingly propose that this maximal activity is exerted by LDH-A tetramer.

## Discussion

Prokaryotic allosteric LDHs were intensively studied, with both their kinetic and structural features inspected in detail. In particular, the occurrence of bacterial enzymes obeying cooperative kinetics and subjected to activation by FBP was reported quite a number of years ago [2]. Remarkably, pyruvate was shown to occupy the allosteric site of bacterial LDHs exposed to solutions devoid of FBP [49,50]. Further, structural studies of the *Lactobacillus casei* LDH have elegantly demonstrated that the transition from the T to the R state of this enzyme can be induced by specific anions (e.g. sulfate), independently of FBP [10]. Among eukaryotic LDHs, representatives from invertebrates were reported to feature allosteric transitions, and to be activated by FBP [51,52]. On the contrary, LDHs from vertebrates are considered to exclusively exert their catalytic action according to Michaelis–Menten kinetics [44]. Nevertheless, quite a number of years ago Fritz reported that the LDH from rabbit skeletal muscle undergoes allosteric transitions when the enzyme activity is assayed in the presence of pyruvate and β-NADH [41]. Moreover, it was recently shown that this enzyme is subjected to allosteric-like large-scale motions [53].

**Figure 10:**
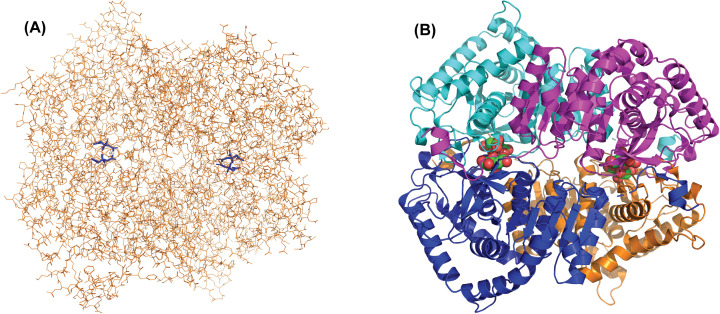
Structure of human LDH-A with citrate bound at the allosteric site The structure of human LDH-A containing four associated citrate molecules (file PDB *5w8i*) is shown. (**A**) The enzyme and citrates are represented with orange lines and blue sticks, respectively. (**B**) The LDH-A subunits are rendered with cyan, magenta, orange, and blue ribbons, and citrates are depicted with green (carbon) and red (oxygen) spheres. The images were obtained with PyMol (The PyMol Molecular Graphics System, version 1.3, Schrödinger, LLC).

We have shown here that human LDH-A behaves as an allosteric enzyme under acidic conditions, and features hyperbolic kinetics at neutral or slightly alkaline pH. In particular, this was observed using both recombinant and natural LDH-A, whose catalytic action was determined to similarly behave as a function of pH. However, we observed that the natural enzyme features significantly higher activity than recombinant LDH-A, with this difference more pronounced under acidic conditions (Figures 1 and 2). This difference, as well as the diversity between the effect exerted by pH on the Hill coefficient of recombinant and natural enzyme, can be ascribed to: (i) post-translational modifications affecting LDH-A catalytic properties; (ii) the presence of ammonium sulfate in assays performed to determine the activity of the natural enzyme (which is provided by the supplier as ammonium sulfate suspension). Concerning the mechanism of sulfate action, we propose that this anion binds between the interfaces of enzyme subunits, with this binding favoring the assembly of LDH-A tetramer, and therefore a higher activity because of the low, if at all, catalytic action exerted by LDH-A dimer and monomer [43]. A similar mechanism is most likely responsible for the higher LDH-A activity triggered by citrate, isocitrate, and malate at both pH 5.0 and 6.5 (Figure 3). Citrate was indeed shown to bind at the subunits interface of human LDH-A (Figure 10), most probably inducing the stabilization of the enzyme tetramer. Interestingly, we observed a peculiar effect triggered by *cis*-aconitate on human LDH-A: this compound does not alter enzyme activity at pH 5.0 (Figure 3A–C), and induces a significant increase in LDH-A catalytic action at pH 6.5 (Figure 3B–D). Taking into account that *cis*-aconitate is rather stable over a pH interval ranging from 5 to 7 [54], it seems unlikely that the pH-dependent effect on LDH-A activity exerted by this tricarboxylic acid (Figure 3) is due to stability features of this enzyme effector. Conversely, it might be that LDH-A is competent in binding *cis*-aconitate at pH 6.5 because of a proper conformation which does not occur at pH 5.0. Accordingly, this would imply a pH-dependent activation of LDH-A by *cis*-aconitate.

Quite a number of years ago, allosteric models relying on protein association–dissociation equilibria were formulated [55,56]. More recently, it was shown that a non-linear dependence of reaction velocity on enzyme concentration is diagnostic of allosteric transitions coupled to dissociation–association equilibria of oligomeric enzymes [57,58]. Moreover, this type of allostery was recognized as responsible for the structural and functional reshaping of oligomeric enzymes, and it was accordingly denoted as the morphein model [58]. Our determinations of reaction velocity as a function of pH and human LDH-A concentration suggest that a consistent dissociation of tetrameric LDH-A occurs over the pH interval inducing enzyme allosteric transitions (Figure 4, cf. Figure 1). These observations are further supported by the DLS experiments reported here, indicating that: (i) tetrameric human LDH-A undergoes dissociation when exposed to solutions the pH of which is lower than 7 (Figure 6 and Table 2); (ii) dilution of the enzyme triggers a consistent dissociation of LDH-A tetramer (Figure 7 and Table 3); (iii) the presence of citrate or NAD^+^/pyruvate counteracts tetramer dissociation (Figure 8 and Table 4). Overall, these outcomes indicate that the allosteric transitions we detected for human LDH-A under acidic conditions are linked to dissociation–association equilibria of the enzyme tetramer. It is also interesting to note that the enzyme activity is maximal at pH 6.5 (Table 1), a condition triggering significant tetramer dissociation.

To investigate how the features of human LDH-A that we detected *in vitro* could eventually affect the generation of lactate *in vivo*, we used HepG2 cells poised at pH 7.3 or 6.5. Surprisingly, we detected a three-fold decrease in the concentration of lactate released by HepG2 cells whose cytosolic pH was lowered to 6.5 (Figure 9). We interpret this observation as due to the dissociation *in vivo* of LDH-A tetramer, with this dissociation induced by the acidic pH.

## Conclusions

We reported here on the allosteric transitions featured *in vitro* by human LDH-A exposed to acidic solutions. The cooperative kinetics featured by LDH-A is the outcome of dissociation–association equilibria of the enzyme tetramer, with pyruvate counteracting dissociation. We also showed that the activity of the enzyme *in vivo* is negatively affected by acidic pH, most likely because of tetramer dissociation. When considering the relevance of LDH-A action in cancer cells, it is interesting to note that the photochemical induction of cytosolic acidification triggered the selective death of cancer hypoxic cells [59]. Accordingly, it is our hope that our observations will prompt further investigations on the biochemical links between human LDH-A cooperative kinetics, cytosolic pH, and cancer cells proliferation.

## Supplementary Material

Supplementary Figures S1-S3

## Data Availability

The data reported in the present study are available from the corresponding author upon request.

## Abbreviations

BCECF: 2′,7′-Bis-(2-carboxyethyl)-5-(6)-carboxyfluorescein, acetoxymethyl ester
DLS: dynamic light scattering
FBP: fructose-1,6-bisphosphate
LDH: lactate dehydrogenase
