# Supplementary material for: Human lactate dehydrogenase A undergoes allosteric transitions under pH conditions inducing the dissociation of the tetrameric enzyme
Source: Biosci Rep. 2022 Jan 28;42(1):BSR20212654. doi: 10.1042/BSR20212654 (PMC8799922; doi:10.1042/BSR20212654)
Supplement: Supplementary Figures S1-S3 [file BSR-2021-2654_supp.pdf]

Supplementary Figure Legends

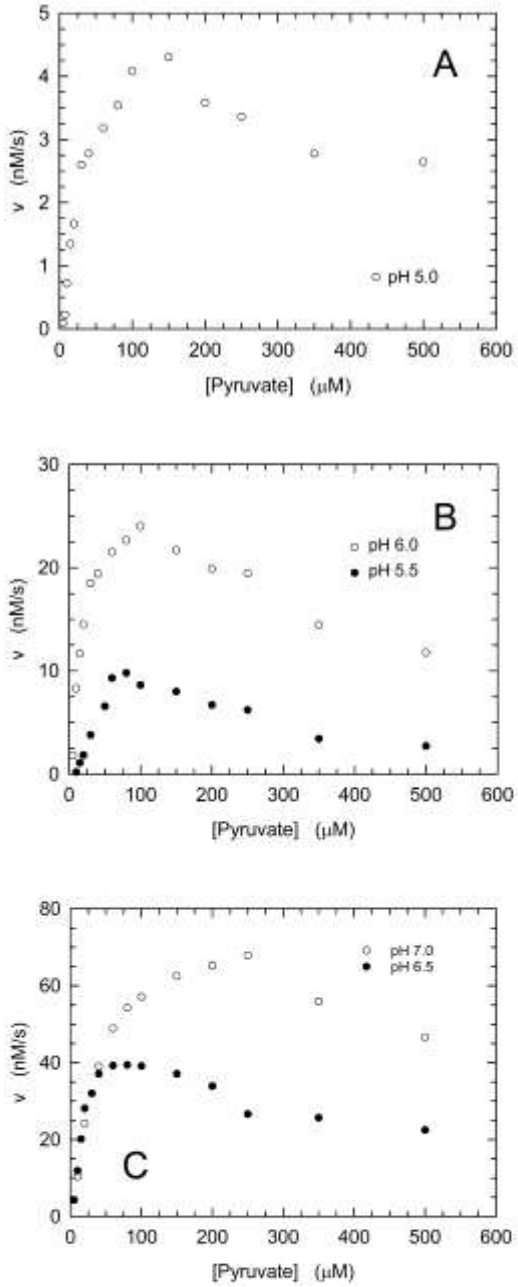

Supplementary Figure S1. Substrate inhibition of recombinant human lactate dehydrogenase.

(A-C) Initial reaction velocities were assayed over a pH interval ranging from 5.0 to 7.0, in the presence of 2.45 nM LDH-A tetramer (9.8 nM subunits), 125 μM β-NADH, and a universal buffer (containing Mes, Mops, and Tris, 25 mM each). The dependence of initial reaction velocities on pyruvate concentration at pH 5.0 (A, empty circles), pH 5.5 and 6.0 (B, filled and empty circles, respectively), pH 6.5 and 7.0 (C, filled and empty circles, respectively), are shown.

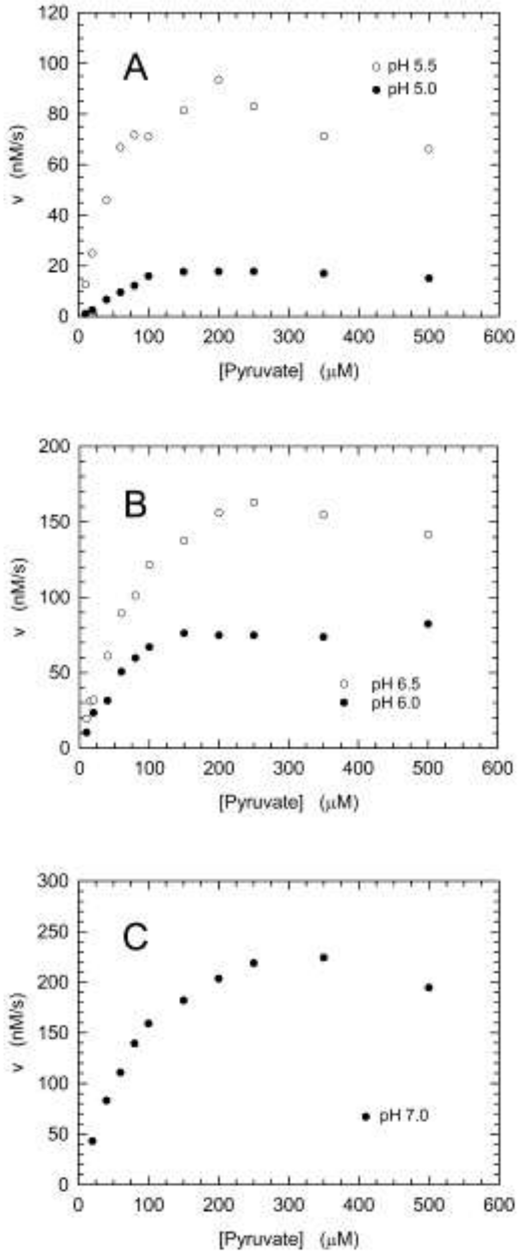

Supplementary Figure S2. Substrate inhibition of natural human lactate dehydrogenase.

(A-C) Initial reaction velocities were assayed over a pH interval ranging from 5.0 to 7.0, in the presence of 1.2 nM LDH-A tetramer (4.8 nM subunits), 125 µM β-NADH, and a universal buffer (containing Mes, Mops, and Tris, 25 mM each). The dependence of initial reaction velocities on pyruvate concentration at pH 5.0 and 5.5 (A, filled and empty circles, respectively), pH 6.0 and 6.5 (B, filled and empty circles, respectively), and pH 7.0 (C, filled circles), are shown.

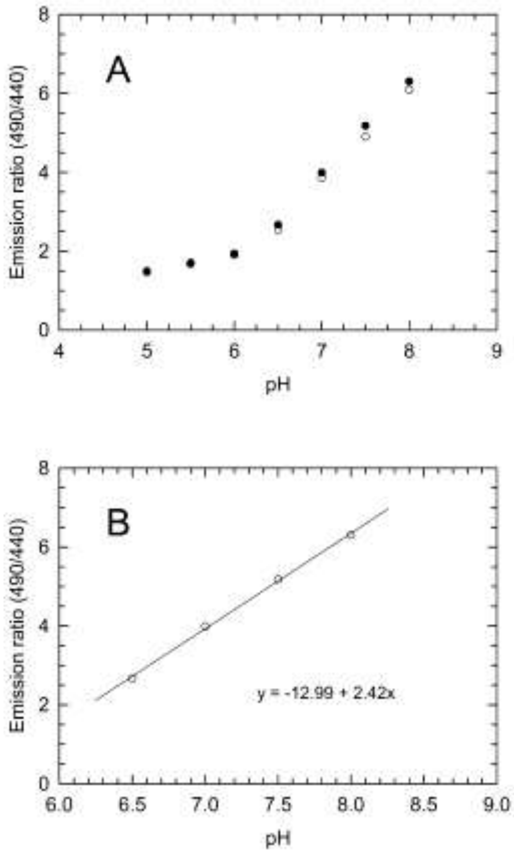

Supplementary Figure S3. Calibration of BCECF fluorescence emission ratio as a function of pH.

(A) Human HepG2 cells were exposed to buffered media (see Methods) equilibrated at pH values ranging from 5.0 to 8.0. After addition of 10 µM nigericin, cells were incubated for 10 minutes at room temperature under mild shaking, and the fluorescence of BCECF was determined exciting samples at 490 or 440 nm, and detecting emission at 535 nm. Two independent measurements for each sample (empty and filled circles) were performed. (B) Calibration curve obtained from the experiment shown in Fig. S3A.
